# Multi-Layer Controls of Cas9 Activity Coupled With ATP Synthase Over-Expression for Efficient Genome Editing in *Streptomyces*

**DOI:** 10.3389/fbioe.2019.00304

**Published:** 2019-11-01

**Authors:** Kai Wang, Qing-Wei Zhao, Yi-Fan Liu, Chen-Fan Sun, Xin-Ai Chen, Richard Burchmore, Karl Burgess, Yong-Quan Li, Xu-Ming Mao

**Affiliations:** ^1^Institute of Pharmaceutical Biotechnology & Research Center for Clinical Pharmacy, The First Affiliated Hospital, School of Medicine, Zhejiang University, Hangzhou, China; ^2^Zhejiang Provincial Key Laboratory for Microbial Biochemistry and Metabolic Engineering, Hangzhou, China; ^3^Zhejiang Provincial Key Laboratory for Drug Evaluation and Clinical Research, Hangzhou, China; ^4^Wolfson Wohl Cancer Research Centre, Institute of Infection, Immunity and Inflammation, University of Glasgow, Glasgow, United Kingdom; ^5^School of Biological Sciences, Institute of Quantitative Biology, Biochemistry and Biotechnology, University of Edinburgh, Edinburgh, United Kingdom

**Keywords:** pleiotropic controls, inducible Cas9 activity, homologous recombination, genome editing, *Streptomyces*

## Abstract

Efficient genome editing is a prerequisite of genetic engineering in synthetic biology, which has been recently achieved by the powerful CRISPR/Cas9 system. However, the toxicity of Cas9, due to its abundant intracellular expression, has impeded its extensive applications. Here we constructed a genetic cassette with triple controls of Cas9 activities at transcriptional, translational and protein levels, together with over-expression of the ATP synthase β-subunit AtpD, for the efficient genome editing in *Streptomyces*. By deletion of *actII-ORF4* in *Streptomyces coelicolor* as a model, we found that constitutive expression of *cas9* had about 90% editing efficiency but dramatically reduced transformation efficiency by 900-fold. However, triple controls of Cas9 under non-induction conditions to reduce its activity increased transformation efficiency over 250-fold, and had about 10% editing efficiency if combined with *atpD* overexpression. Overall, our strategy accounts for about 30-fold increased possibility for successful genome editing under the non-induction condition. In addition, about 80% editing efficiency was observed at the *actII-ORF4* locus after simultaneous induction with thiostrepton, theophylline and blue light for Cas9 activity reconstitution. This improved straightforward efficient genome editing was also confirmed in another locus *redD*. Thus, we developed a new strategy for efficient genome editing, and it could be readily and widely adaptable to other *Streptomyces* species to improve genetic manipulation for rapid strain engineering in *Streptomyces* synthetic biology, due to the highly conserved genetic cassettes in this genus.

## Introduction

Given the high efficiency and precise targeting, CRISPR/Cas9 is regarded as the most powerful genome editing toolkit for dissection of molecular mechanisms and control of gene expression (Xu and Qi, 2018). Particularly, it has exhibited great potential in synthetic biology for cell reprogramming, protein engineering and circuitry design (Fellmann et al., 2017; Ho and Chen, 2017), and also for microbial metabolic engineering and manipulation of genetically intractable microorganisms (Cho et al., 2018b; Shapiro et al., 2018).

However, the off-target effects and toxic activity of Cas9 have been observed, due to excessive production of Cas9, along with non-specific guiding by sgRNA, for non-specific binding and cleavage of non-target DNA (Reisch and Prather, 2015; Cui and Bikard, 2016; Cho et al., 2018a). Meanwhile, many efforts have been taken to reduce Cas9 toxicity by controlling its endogenous nuclease activity (Richter et al., 2017). These include transient delivery of Cas9/sgRNA ribonucleoprotein to avoid over-accumulation of intracellular Cas9 during cell propagation (DeWitt et al., 2017), and chemically-inducible expression of Cas9 to reduce off-target cleavages and facilitate precise genome editing (Cao et al., 2016). Moreover, post-translational control of Cas9 by fusion of Cas9 to ERT2 (Liu et al., 2016) or Cas9 splits to FKBP and FRB (Zetsche et al., 2015), respectively, can sequester Cas9 in the cytoplasm, and trans-localization of Cas9 into nuclei for genome editing is efficiently induced by 4-hydroxytamoxifen (Liu et al., 2016) or rapamycin (Zetsche et al., 2015).

The visible consequence of Cas9 toxicity is the drastic reduction of transformant number during introduction of genetic elements into cells, as already observed in *Escherichia coli* (Cui and Bikard, 2016), yeast (DiCarlo et al., 2013), and *Streptomyces* (Tong et al., 2015; Zeng et al., 2015; Cao et al., 2016), and most often results in failure of genetic manipulations. *Streptomyces* is a genus of soil-dwelling filamentous bacteria rich in gene clusters and production of clinically used pharmaceuticals (Kieser et al., 2000), and synthetic biology has been highly developed for their precious natural products (Palazzotto et al., 2019). However, this formidable obstacle will severely impede efficient genetic development of the rich reserves of natural products, particularly in some newly isolated species. Though CRISPR/Cas9 toolkits with *cas9* expressed under a thiostrepton-inducible promoter *tipAp* (Huang et al., 2015; Cao et al., 2016) or constitutive promoters (Cobb et al., 2015; Zeng et al., 2015) have been reported in this genus, the genome editing system should be improved for more efficiency.

Recently in *Streptomyces*, a theophylline-dependent riboswitch has been developed (Horbal and Luzhetskyy, 2016). An 85-bp riboswitch can reduce downstream protein expression to an extremely low level, but several hundred-fold induction was observed when the inducer is added, suggesting that it is an ideal tool for the translational control. Moreover, another version of split Cas9 system—blue light inducible Cas9 reconstitution—has been developed. The fungal photo-receptor Vivid is engineered into two proteins, positive Magnet (pMag) and negative Magnet (nMag), which hetero-dimerize upon exposure to blue light and have switch-off kinetics by four orders of magnitude (Kawano et al., 2015). This photo-switchable system allows development of an photo-activatable split Cas9 (N713-pMag and nMag-C714) for the precise controls of Cas9 activity for gene editing (Nihongaki et al., 2015).

DSB (double-strand breakage) caused by Cas9 should be precisely repaired through homology directed recombination (HDR), which is exerted by the ATP-dependent DNA repair system. ATP plays important and essential roles in HDR, as it is associated with recombinase (RecA/Rad51) filaments on DNA, and its hydrolysis is essential for the dynamic interaction between RecA-ssDNA (Reymer et al., 2015), RecA sliding along DNA (Kim et al., 2017), and conformational change during Rad51 filament disassembly (Brouwer et al., 2018).

Here we constructed a genetic cassette for efficient genome editing in *Streptomyces* by pleiotropic controls of Cas9 activity at transcription, translation and protein levels, combined with overexpression of ATP synthase β-subunit AtpD. Using *Streptomyces coelicolor* as a model, this strategy has dramatically enhanced the transformation efficiency by about 250-fold and increased nearly 30-fold probability to obtain mutants over the traditional CRISPR/Cas9 system under the non-induction condition. Moreover, about 80% deletion rate was observed at the *actII-ORF4* locus in the unedited transformants when Cas9 activity was induced and reconstituted. In addition, this strategy by uncoupling DNA introduction and Cas9-mediated DNA cleavage to improve the chance of genome editing was also verified at the *redD* locus in *S. coelicolor*. Thus, here we provide an efficient genetic tool for rapid genome editing in *S. coelicolor*, and it could be also widely and readily applied in other *Streptomyces* species.

## Materials and Methods

### Strains and Media

*Streptomyces coelicolor* M145 (Kieser et al., 2000) and a daptomycin producer *Streptomyces roseosporus* SW0702 (Mao et al., 2015) were used for genome editing. *E. coli* DH5α (Merck) was used as a general plasmid cloning host. *E. coli* ET12567/pUZ8002 was used to introduce plasmids into *Streptomyces* by inter-species conjugation. *E. coli* strains were cultured in LB medium. Liquid 3% TSB plus 5 % PEG6000 was used for vegetative growth of *Streptomyces* for conjugation after inoculum of spores. Solid R2YE was used as the medium for *S. coelicolor* to produce actinorhodin (Act) and undecylprodigiosin (Red), antibiotic sensitivity assays (with 50 μg/ml apramycin) and also for colony PCR, while MSF medium was for spore preparation and conjugation (Kieser et al., 2000).

### Plasmid Construction

All the primers and plasmids used in this work are listed in Tables S1, S2, respectively, and the detailed procedures for plasmid construction were described in Supplementary Materials and Methods. Chemically synthesized DNA sequences of pMag and nMag were shown in Figures S28, S29, respectively.

### Colony Counting

For plates with <100 transformants, the total colonies were counted directly. For conjugations expected with over 100 transformants according to the preliminary experiments, the bacterial mixtures were plated in 1: 15 or 1: 3 dilution in triplicate. Each plate was divided into eight equal parts, and only one representative part was counted. The total transformants were estimate based on counting from each plate, and shown as the mean ± SD (standard deviation) from three independent transformations.

### Genotype Validation by Colony PCR

The transformants on the MSF medium were patched on the R5 medium containing 50 μg/ml apramycin, and further cultured at 30°C for 3 days. The genotypes of transformants were verified by colony PCR with 2 × Taq Plus Master Mix (Dye Plus) (Vazyme), as very little mycelia were transferred to the PCR reactions, and lysed by heating at 95°C for 5 min before PCR. Primers 46 + 47 and 48 + 49 were used for confirmative PCR of *actII-ORF4* deletion, while primers 50 + 51 and 52 + 53 for confirmation of *redD* deletion.

### Induction of the Reconstituted Cas9

The spores of *S. coelicolor* strains containing the inducible Cas9 cassette and overexpressed *atpD* were inoculated in TSB + PEG6000, and cultured in a cell-tissue bottle for more transmissibility and proximity of blue light. Cas9 activity was induced with the combination of inducers, including 5 μg/ml thiostrepton, 4 mM theophylline and blue light irradiation (470 ± 20 nm LED light source) with a power of 6.4 W and distance of 10 cm. After induction for 5 days, the mycelia were streaked on R2YE medium for genotype verification by colony PCR.

## Results

### Design of an Inducible Split-Cas9 Genetic Cassette

Constitutively over-expressed *cas9* is proposed to be toxic to many microbial species in genome editing, as shown by the drastic reduction in the number of transformants (DiCarlo et al., 2013; Zeng et al., 2015; Cao et al., 2016; Cui and Bikard, 2016). To overcome this problem, we set out to control intracellular Cas9 activity at transcriptional, translational and protein levels. Our hypothesis is that the reduced Cas9 activity should be beneficial for cell viability during transformation, and subsequently efficient genome editing would occur after Cas9 activity is induced for DSB coupled with HDR.

**Figure 1:**
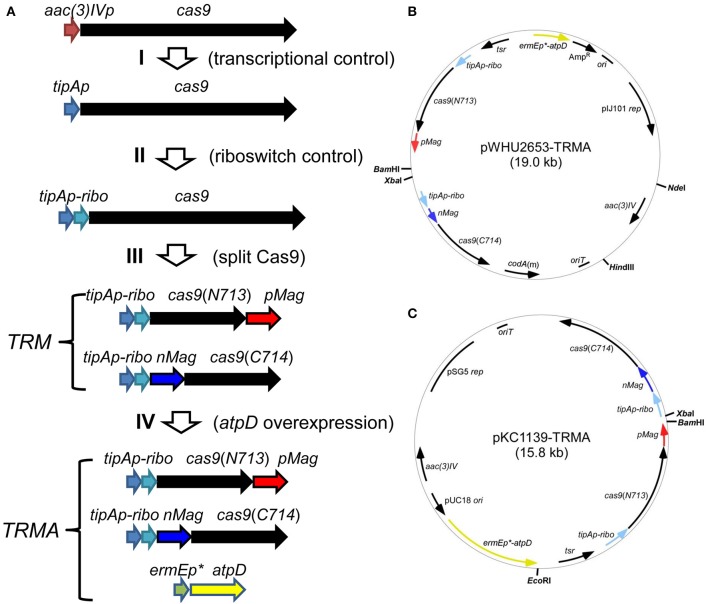
Engineered controls of Cas9 activities and ATP supply for genome editing. **(A)** Strategy for the stepwise controls of Cas9 under the expression of *tipAp*, riboswitch and blue light for the pleiotropic controls at transcriptional (I), translational (II), and protein (III) levels. The ATP synthase gene *atpD* from *S. coelicolor* was additionally overexpressed under ermEp* (IV). **(B,C)** Two vectors containing genetic cassettes based on pIJ101 *ori* (from pWHU2653) and pSG5 *ori* (pKC1139) were shown as pWHU2653-TRMA **(B)** and pKC1139-TRMA **(C)**, respectively. All the genetic cassettes were labeled as in **(A)** and multiple-cloning sites are shown in bold.

For this purpose, we developed an inducible genetic cassette to control the Cas9 activity in *Streptomyces*. The codon optimized Cas9 (from *Streptococcus pyogenes*) in the plasmid pWHU2653 was put under the control of an inducible promoter *tipAp* instead of the constitutive *aac(3)IV* promoter [*aac(3)IVp*] (Huang et al., 2015; Zeng et al., 2015). Subsequently, a theophylline-inducible riboswitch was inserted after *tipAp* for the translational control (Rudolph et al., 2015; Horbal and Luzhetskyy, 2016). In addition, Cas9 was split expressed as N713-pMag and nMag-C714 fusions to control Cas9 activity at the protein level (Nihongaki et al., 2015). Moreover, since HDR is highly dependent on energy, so *atpD*, encoding the β-subunit of ATP synthase from *S. coelicolor* (SCO5373), which is the catalytic subunit for ATP synthesis, was expressed under a strong constitutive promoter ermEp^*^ (Figure 1).

### Genome Editing Under Non-induction Conditions

**Figure 2:**
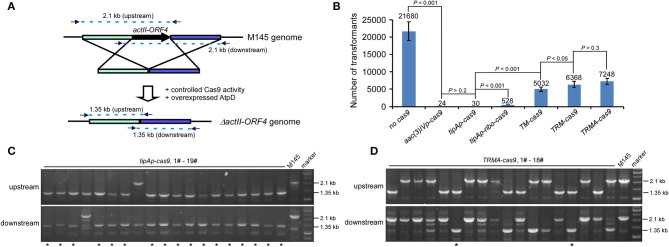
*actII-ORF4* editing in *S. coelicolor* with fine-tuned controls of Cas9 and overexpression of *atpD* on the pIJ101 *ori* plasmid under non-induction conditions. **(A)** Diagram of *actII-ORF4* deletion in *S. coelicolor* based on engineered controls of Cas9. The primers for diagnostic PCR were shown as arrows, and the predicted sizes of PCR fragments in wild type (M145) and the Δ*actII-ORF4* mutant were also shown. The two 2.1 kb fragments are the predicted upstream and downstream PCR products at *actII-ORF4* locus of wild type, while the two 1.35 kb fragments are the corresponding PCR products after *actII-ORF4* is deleted in the mutant. Desired genome editing is verified by simultaneous genotype investigation of the upstream and downstream regions by PCR. **(B)** Transformation efficiency during *actII-ORF4* deletion in *S. coelicolor* with various combinatory controls of Cas9 activities based on the pIJ101 *ori* plasmid. The estimated transformants were shown as mean ± SD (standard deviation) from three independent transformations, and the average transformant numbers were shown on the corresponding columns. *ribo*, theophylline-inducible riboswitch; *TM*, both Cas9 N713-pMag and nMagHigh1-Cas9 C714 split fusions expressed under *tipAp*; *TRM*, both Cas9 N713-pMag and nMagHigh1-Cas9 C714 splits expressed under *tipAp* and riboswitch; *TRMA*, overexpressed *atpD* along with *TRM*. Statistical significance (*P* < 0.001 or *P* < 0.05) was determined by two-tailed unpaired Student's test. **(C,D)** Diagnostic PCR for CRISPR/Cas9-based *actII-ORF4* deletion with Cas9 expressed from *tipAp* **(C)** or with engineered control of Cas9 **(D)** as shown in Figure 1A. The successfully edited mutants were indicated with asterisks.

To test our above hypothesis, we attempted to delete *actII-ORF4* in *S. coelicolor* M145 as a proof of concept, since this gene has been shown to have 100% editing efficiency if *cas9* is expressed from *tipAp*. Deletion of *actII-ORF4* disrupted the production of the blue pigment actinorhodin (Act) (Huang et al., 2015). The spacer sequence and homologous arms were the same as described previously (Figure 2A; Huang et al., 2015). High transformation efficiency was observed without Cas9 (>21,000 transformants), but only about 24 or 30 transformants appeared if *cas9* was expressed under *aac(3)IVp* or *tipAp* (Figure 2B, Figure S1). However, further introduction of riboswitch (*tipAp-ribo-cas9*) and split Cas9 (*TM-cas9*) under *tipAp* significantly enhanced transformants to about 530 and 5000, which was about 18 (528/30) and 168 (5032/30) -fold higher than the control *tipAp-cas9*, respectively. Moreover, about 6400 transformants were obtained if riboswitch and split fusion expression were combined to control Cas9 activity (*TRM-cas9*) (Figure 2B, Figure S1). These results suggested that reduction of Cas9 activity at translational and protein levels could significantly improve transformation.

Diagnostic PCR data showed that no editing at *actII-ORF4* was observed if Cas9 was not included (Figure S2). High efficiency (about 90%) of *actII-ORF4* deletion was found when *cas9* was expressed from *aac(3)IVp* or *tipAp* (Figure 2C, Figure S3), though very few transformants were observed (Figure 2B). However, if *cas9* was controlled under both riboswitch and split proteins (*TRM-cas9*), no successful deletion was observed. Instead, most transformants showed single cross-over integration of the plasmid into the genome (Figure S4), though high transformation efficiency was observed.

Our above data suggested that reduced Cas9 toxicity by pleiotropic controls of Cas9 activity significantly improved cell viability during transformation, but no genome editing was observed. However, leakage effects have been reported both from riboswitch and split Cas9 under non-induction conditions (Kawano et al., 2015; Nihongaki et al., 2015; Rudolph et al., 2015; Horbal and Luzhetskyy, 2016), suggesting that Cas9 could have residual activity *in vivo*, thus causing DSB. We speculated that genome editing to some extents might occur if we could enhance the efficiency of HDR after Cas9-caused DSB.

The bacterial recombinase RecA for HDR is highly conserved among *Streptomyces* species (Figures S5, S6). It is anticipated that increased RecA concentration would promote recombination after DSB to support microbial survival (Prentiss et al., 2015). However, our data showed that over-expression of *recA* from *S. coelicolor* (SCO5769) dramatically reduced transformation efficiency in about 60% (6,400–2,700 transformants) (Figure S7), and very few plasmid integrations (only 1/19 cross-over) were observed (Figure S8). These data suggested that RecA has a negative role in DSB repair after Cas9 cleavage in *Streptomyces*. However, over-expression of AtpD, the β-subunit of *Streptomyces* F1F0-type ATP synthase (Capaldi and Aggeler, 2002), did not significantly influence the transformation efficiency (6,400–7,200 transformants) (*TRMA-cas9*) (Figure 2B), and interestingly, about 10% genome editing efficiency was observed (2/18) (Figure 2D). These data suggested that elevation of *aptD* expression promotes HDR on Cas9-caused DSB. Again, further introduction of RecA reduced over half transformation (Figure S7) and diminished genome editing even with over-expressed AtpD (Figure S9). Thus, based on our editing percentage from *TRMA-cas9* transformation, it was estimated that over 700 mutants would be obtained, which was over 30 (700/24^*^90%)-fold higher in chance than the constitutively expressed *cas9* [*aac(3)IVp-cas9*].

To further confirm our hypothesis, another gene *redD* was chosen for editing with this engineered CRISP/Cas9 system (Figure S10), together with the same homologous arms and spacer used in a previous study (Huang et al., 2015). Consistent with the results from *actII-ORF4* editing, over 500-fold reduction of transformation efficiency was observed if Cas9 was constitutively expressed from *aac(3)IVp* (48 colonies) or *tipAp* (36 colonies) compared to the control without Cas9 (about 27,000 transformants), while introduction of riboswitch, Mag-based split proteins or both, for Cas9 activity control increased transformation efficiency with about 17-fold (840/48), 100-fold (4860/48), or 115-fold (5520/48) compared to Cas9 expressed under *aac(3)IVp* (Figure S11). Diagnostic PCR showed that no genome editing was observed without Cas9 (Figure S12), and Cas9 expressed from *aac(3)IVp* conferred <10% (1/12) editing efficiency, though most strains showed integrative forms of the plasmid (Figure S13), consistent with the previous report of <30% *redD* editing efficiency (Huang et al., 2015). After Cas9 was controlled under riboswitch and the split form (*TRM-cas9*), most transformants displayed replicative forms of the plasmid (Figure S14). However, further over-expression of AtpD promoted integration of the plasmid and 8.6% (2/23) editing efficiency was obtained (Figure S15), which could be used for estimation that about 475 mutants would be obtained. The data from genome editing at the *redD* locus suggested that our strategy had over 100-fold [475/48^*^(1/12)] higher possibility than the traditional method with constitutively expressed *cas9* to obtain the genetic modified strains (Figures S11, S15).

All our above results suggested that reduced intracellular Cas9 activity by pleiotropic controls is significantly beneficial for cell viability during transformation, and resulted in a higher chance to obtain the desired genome-edited mutants if combined with over-expressed AtpD, while the recombinase RecA is detrimental to HDR after Cas9-mediated DSB possibly by prevention of efficient plasmid integration, since the toxicity from over-expressed RecA has been observed in a closely related species *S. lividans* (Vierling et al., 2000).

### Engineered CRISPR/Cas9 System on a Temperature-Sensitive Backbone

Here we engineered the CRISPR/Cas9 system on the plasmid pWHU2653, which contains a counter-selection marker *codA* for plasmid loss in the presence of 5-FC (Zeng et al., 2015). However, this selection system is not always suitable in some *Streptomyces* species with high resistance (Dubeau et al., 2009), and we observed that the loss rate of this plasmid was low (about 4%) without the selection pressure of 5-FC (Figure S16). Thus, we developed the same CRISPR/Cas9 system in a *Streptomyces* temperature-sensitive but multiple-copy plasmid pKC1139 (Bierman et al., 1992), which will be readily lost over 34°C and therefore advantageous for multiple cycles of genome editing (Figure 1C).

**Figure 3:**
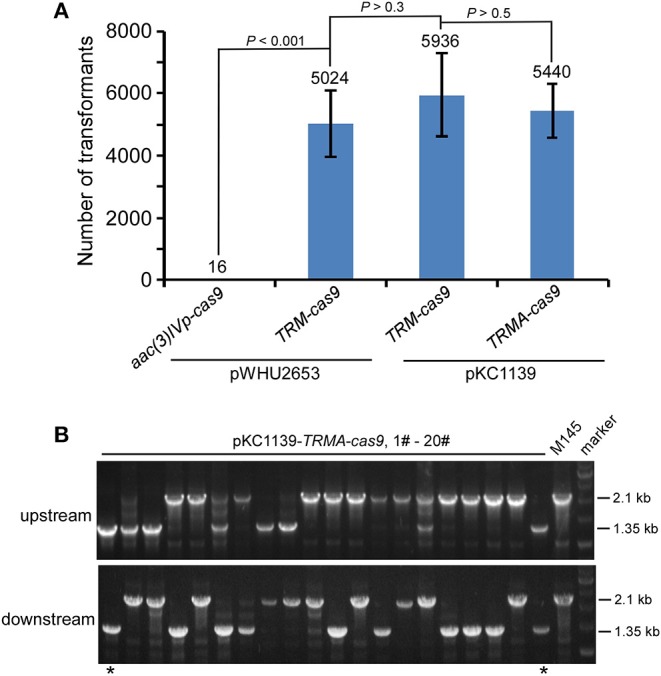
*actII-ORF4* deletion in *S. coelicolor* with engineered Cas9 and *atpD* overexpression from the pSG5 *ori* plasmid under non-induction conditions. **(A)** Transformation efficiency for *actII-ORF4* deletion in *S. coelicolor* with engineered controls of Cas9 activities as in Figure 2B, but based on the pSG5 *ori* plasmid. The estimated transformants were shown as mean ± SD (standard deviation) from three independent transformations, and the average transformant numbers were shown on the corresponding columns. Statistical significance (*P* < 0.001) was determined by two-tailed unpaired Student's test. **(B)** Diagnostic PCR for *actII-ORF4* deletion based on the same strategy as Figure 2D, but with pSG5 *ori* on the pKC1139 plasmid. The successfully deleted strains were indicated with asterisks.

We found that this engineered CRISPR/Cas9 system on pKC1139 conferred comparable transformation efficiency as on pWHU2653, with or without over-expressed AtpD (Figure 3A, Figure S17). Consistent with results shown above, although residual Cas9 activity was still observed, no genome editing was detected even on this multiple-copy plasmid backbone without over-expressed AtpD, as exemplified by deletion of *actII-ORF4* (Figure S18) or *redD* (Figure S19) in *S. coelicolor*. Estimated 10% (2/20) and 8.3% (1/12) editing efficiency was found on *actII-ORF4* and *redD* loci (Figure 3B, Figure S20), respectively, when *atpD* was further over-expressed. As expected, the plasmid was lost at a rate of 100% (30/30) at the restricted temperature (37°C) (Figure S21).

### Efficient Genome Editing Under Induction Conditions

**Figure 4:**
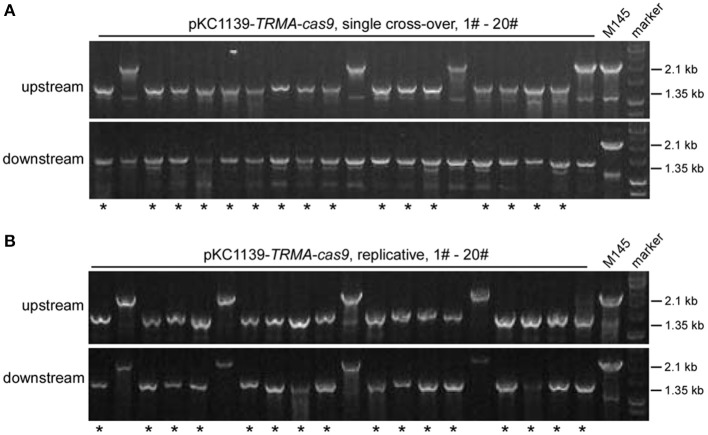
*actII-ORF4* deletion in *S. coelicolor* with engineered Cas9 and *atpD* overexpression from the pSG5 *ori* plasmid under induction conditions. The unedited transformants containing pKC1139-TRMA-cas9 in the single cross-over **(A)** or replicative form **(B)** from Figure 3B were simultaneously induced with thiostrepton, theophylline and blue light, and the genotype of twenty single colonies was investigated with diagnostic PCR as in Figure 2A. The successfully deleted strains were indicated with asterisks.

Most transformants remained genetically intact with triple-layer controlled Cas9 (Figures 2, 3). Then we investigated whether the genome could be further edited upon induction of Cas9 activity. We found that the transformants with pKC1139-TRMA-cas9, either in single cross-over or replicative form, were not genetically edited at the *actII-ORF4* locus if only induced with chemicals (Tsr and Theo) or the blue light (Figures S22, S23). However, when all inducers were included, efficient genome editing (80% = 16/20) was observed with both forms (Figure 4). Phenotypic analysis of actinhordin (Act) biosynthesis in *S. coelicolor* also confirmed that all the Δ*actII-ORF4* mutants failed to produce this blue pigment, regardless of plasmid backbones and induction/non-induction conditions (Figure S24), suggesting that they were all successfully edited. The similar results were obtained on the *redD* locus, as genome editing was only observed after induction with both chemicals and blue light (Figures S25–S27), but 35% (7/20) and 40% (8/20) efficiency were only detected from single cross-over and replicative plasmids, respectively (Figure S27), which might result from the low efficiency of genome editing at this locus (Huang et al., 2015).

## Discussion

Toxicity from the constitutively active Cas9 will dramatically reduce the transformation efficiency, and most often, no transformant at all will be obtained, thus obstructing efficient genetic manipulations for desired genome editing (DiCarlo et al., 2013; Zeng et al., 2015; Cao et al., 2016; Cui and Bikard, 2016). In these cases, introduction of genetic cassettes into cells and subsequent genome editing occur simultaneously at one step. Here we demonstrated an alternative strategy to uncouple transformation and genome editing in two steps, and showed that it can be applied straightforwardly for efficient genome editing in *Streptomyces*.

In this work, a genetic cassette was developed for multi-layer controls of Cas9 activity at transcriptional, translational and protein levels. These include thiostrepton-inducible *tipAp*, the theophylline-inducible riboswitch (Rudolph et al., 2015) and the blue light-inducible Cas9 reconstitution system (Nihongaki et al., 2015) to reduce intracellular Cas9 toxicity. Combination of these two latter cassettes has the best effects with over 250-fold increased transformation. Further over-expression of ATP synthase resulted in about 10% editing efficiency. Though not as high in genome editing as the traditional method with constitutively expressed *cas9* (Huang et al., 2015; Zeng et al., 2015), our new strategy is estimated to enhance about 30-fold of chance to obtain the genetically edited mutants. Moreover, it is encouraging that subsequent reconstitution of Cas9 activity in the unedited transformants showed 40–80% editing efficiency, probably depending on the genomic environments or the 20-bp spacer sequences.

Here in the well-studied model *Streptomyces* species *S. coelicolor*, the transformation efficiency dropped about 900-fold (21680/24 = 903) with constitutively expressed *cas9* (Figure 2B), and far <100 transformants could be obtained, though the genome editing efficiency was almost 100%. This similar observation was also reported previously (Zeng et al., 2015). However, in some genetically intractable industrial or newly identified *Streptomyces* strains, the efficient conjugation is not well-established. The low transformation efficiency in these strains will most often result in null transformant with overexpressed Cas9 and significantly delay the study of microorganisms themselves, as well as strain improvement or development. Our strategy by uncoupling of transformation and genome editing can potentially enhance the chance to obtain the transformants, and make it sure to successfully edit the strains subsequently after induction. Though more time might be needed than the traditional Cas9 expression cassette if transformants could be obtained, our strategy will be more efficient than the traditional gene knock-out methods in *Streptomyces* solely depending on homologous recombination, such as pKC1139-based gene deletion (Bierman et al., 1992) and PCR-targeting (Gust et al., 2003). In addition, the theophylline-inducible riboswitch and the blue light-inducible pMag/nMag cassettes could have wide applicability in *Streptomyces*, suggesting that the fine-tuned controls of Cas9 activity will be readily adaptable in other *Streptomyces* species. This idea was further confirmed by our data on a daptomycin producer *Streptomyces roseosporus*, in which a putative daptomycin-producing related gene *dptP* was used as a model (Miao et al., 2005). Constitutive expression of *cas9* again reduced transformation efficiency for over 1000-fold, while introduction of stepwise combination of the regulatory genetic elements (*tipAp*, riboswitch, and Mag proteins) to reduce Cas9 activity could increase transformation efficiency (Figure S30). This wide feasibility of this genetic system holds a highly promising application in synthetic biology of *Streptomyces*, and will benefit the microbiologists for efficient genome editing and industry for rapid strain improvement.

## Data Availability Statement

All datasets generated for this study are included in the article/Supplementary Material.

## Author Contributions

KW, Y-QL, and X-MM have conceived the project. KW, Q-WZ, Y-FL, C-FS, X-AC, Y-QL, and X-MM have designed experiments. KW, Q-WZ, Y-FL, and C-FS have demonstrated the experiments. All authors have analyzed the data, prepared the manuscript, and approved the final version.

### Conflict of Interest

The authors declare that the research was conducted in the absence of any commercial or financial relationships that could be construed as a potential conflict of interest.
