# Supplementary material for: Multi-Layer Controls of Cas9 Activity Coupled With ATP Synthase Over-Expression for Efficient Genome Editing in Streptomyces
Source: Front Bioeng Biotechnol. 2019 Nov 1;7:304. doi: 10.3389/fbioe.2019.00304 (PMC6839703; doi:10.3389/fbioe.2019.00304)
Supplement: Supplementary file 1 [file Data_Sheet_1.PDF]

Supplementary Material

1 Supplementary Materials and Methods

Plasmid construction

For expression of Cas9 under *tipAp* and riboswitch, both plasmids pWHU2653 (1) and pET28a (Merck, Germany) were digested with *Eco*RI/*Xba*I, and the 4.7 kb fragment containing cas9-gRNA was ligated to the 5.2 kb fragment to plasmid pET28a-cas9-gRNA. Fragment *tsr-to-tipAp* was amplified with primers 1 + 2 from plasmid pIJ8600 (2), and fused to *Sac*I/*Not*I digested pET28a-cas9-gRNA by Gibson assembly with ClonExpress MultiS One Step Cloning Kit (Vazyme, China) to give rise to pET28a-tipAp-cas9-gRNA. It was then digested with *Eco*RI/*Bgl*II, and the 6.3 kb fragment was ligated to the 9.4 kb fragment digested from pWHU2653 with *Eco*RI/*Bam*HI to plasmid pWHU2653-tipAp. A 114 bp fragment containing the riboswitch was *in vitro* amplified with primers 3 + 4 + 5, and fused to *Nde*I-digested pET28a-tipAp-cas9-gRNA by Gibson assembly to create plasmid pET28a-tipAp-ribo, which was then digested with *Eco*RI/*Bgl*II and ligated to 9.4 kb fragment digested from pWHU2653 above for the plasmid pWHU2653-tipAp-ribo.

For split expression of Cas9, both DNA fragments for pMag and nMagHigh1 (nMag) were chemically synthesized (Genscript, China) according to the reference (3) with a linker GGSGSSGGSG at the N-terminus of pMag and a linker GGSGGSGGGSGSGSGG at the C-terminus of nMag, and cloned into the vector pTA2 (Toyobo, Japan). Detailed DNA sequences with codon optimized for *Streptomyces* were shown in Fig. S28 and S29. Fragment *to-tipAp* containing the *Xba*I/*Nde*I site was amplified from pIJ8600 with primers 6 + 7, and fused to the *Xba*I site of pTA2 by Gibson assembly for plasmid pTA2-tipAp. Then nMag and Ccas9 (C-terminal 1965 bp of *cas9*) were amplified with primers 8 + 9, and primer 10 + 11 from pTA2-nMag and pWHU2653, respectively, and fused to pTA2-tipAp digested with *Nde*I by Gibson assembly for plasmid pTA2-tipAp-nMag-Ccas9. Fragments Ncas9 (N-terminal 2136 bp of *cas9*) and pMag were amplified with primers 12 + 13, primers 14 + 15 from pWHU2653 and pTA2-pMag, respectively, and fused to *Nde*I/*Nhe*I-digested pET28a-tipAp-cas9-gRNA for plasmid pET28a-tipAp-Ncas9-pMag. This plasmid was further digested with *Xba*I/*Bgl*II, and ligated to the tipAp-nMag-Ccas9 fragment from pTA2-tipAp-nMag-Ccas9 digested with *Xba*I/*Bgl*II for plasmid pET28a-tipAp-Mag-cas9. Finally, this plasmid was digested with *Eco*RI/*Bgl*II and ligated to the 9.4 kb *Eco*RI/*Bam*HI fragment from pWHU2653 to the plasmid pWHU2653-TM-cas9.

For further expression of split Cas9 under the control of riboswitch, the 114 bp fragment above was fused to *Nde*I-digested pET28a-tipAp-Ncas9-pMag and pTA2-tipAp-nMag-Ccas9, respectively, for pET28a-tipAp-ribo-Ncas9-pMag and pTA2-tipAp-ribo-nMag-Ccas9 by Gibson assembly. Then pTA2-tipAp-ribo-nMag-Ccas9 was digested with *Xba*I/*Bgl*II for the fragment tipAp-ribo-nMag-Ccas9, and ligated to *Xba*I/*Bgl*II digested pET28a-tipAp-ribo-Ncas9-pMag for the plasmid pET28a-tipAp-ribo-Mag-cas9, which was further digested with *Eco*RI/*Bgl*II. The 8.6 kb fragment was ligated to the 9.4 kb *Eco*RI/*Bam*HI fragment from pWHU2653 to the plasmid pWHU2653-TRM-cas9.

For combined ectopic expression of *recA* and *aptD*, the constitutive promoter *ermEp** and *recA* were amplified from pLM1 (4) and the genomic DNA of *S. coelicolor* M145 with primers 16 + 17, and primers 18 + 19, respectively, and fused to the *Nde*I site of pWHU2653-TRM-cas9 for plasmid

pWHU2653-TRM-cas9-recA. Moreover, *ermEp** and *atpD* were amplified from pLM1 and *S. coelicolor* M145 genome with primers 20 + 21, and primers 22 + 23, respectively, and fused to the *Eco*RI site of pWHU2653-TRM-cas9 for plasmid pWHU2653-TRMA-cas9, and *Eco*RI site of pWHU2653-TRM-cas9-recA for the plasmid pWHU2653-TRMA-cas9-recA.

To replace the pIJ101 *ori* in pWHU2653 with pSG5 *ori* from pKC1139 (5), the 8.0 kb fragment from *Eco*RI/*Bgl*II-digested pWHU2653-TRM-cas9 was ligated to the *Eco*RI/*Bgl*II-digested 6.0 kb fragment from pKC1139 for the plasmid pKC1139-TRM-cas9. *ermEp** and *atpD* were amplified from pLM1 and *S. coelicolor* M145 genome with primers 24 + 21, and primers 22 + 25, respectively, and fused to the *Eco*RI site of pKC1139-TRM-cas9 by Gibson assembly for the plasmid pKC1139-TRMA-cas9.

For plasmids in genome editing at *actII-ORF4* and *redD* loci, primers 26 + 27 and primers 28 + 29 were annealed as the spacers and ligated to the *Bae*I site of pWHU2653, pWHU2653-tipAp, pWHU2653-tipAp-ribo, pWHU2653-TM-cas9, pWHU2653-TRM-cas9, pWHU2653-TRM-cas9-recA, pWHU2653-TRMA-cas9 and pWHU2653-TRMA-cas9-recA, respectively. The homologous regions for HDR were amplified with primers 30 + 31 for *actII-ORF4* upstream arm and primers 32 + 33 for *actII-ORF4* downstream arm, primers 34 + 35 for *redD* upstream arm and primers 36 + 37 for *redD* downstream arm from *S. coelicolor* M145 genome, and fused to the *Hin*dIII site of above plasmids by Gibson assembly following spacer insertion. To remove *cas9* as a negative control, primers 38 + 39 were annealed and ligated to *Eco*RI/*Nhe*I digested pWHU2653-actII-ORF4 and pWHU2653-redD, respectively, to pWHU2653-actII-ORF4-cas9 and pWHU2653-redD-cas9. For plasmids with pSG5 *ori*, the *actII-ORF4* spacer was ligated to the *Bae*I site of pKC1139-TRM-cas9 and pKC1139-TRMA-cas9, respectively. The *actII-ORF4* upstream arm was amplified with primers 40 + 31, while two *actII-ORF4* downstream arms were amplified with primers 32 + 41 and primers 32 + 42. The upstream arm combined with the first downstream arm was fused to pKC1139-TRM-cas9-spacer for plasmid pKC1139-TRM-cas9-actII-orf4, while the upstream arm combined with the second downstream arm was fused to pKC1139-TRMA-cas9-spacer for plasmid pKC1139-TRMA-cas9-actII-orf4. The *redD* editing plasmids were constructed in the same procedure, except that primers 43 + 35 was for the upstream homologous arm, and primers 36 + 44 and 36 + 45 for the two downstream arms, for the plasmids pKC1139-TRM-cas9-redD and pKC1139-TRMA-cas9-redD.

For plasmids of genome editing at *dptP* locus in *Streptomyces roseosporus*, primers 54 + 55 were annealed as the spacers and ligated to the *Bae*I site of pWHU2653, pWHU2653-tipAp, pWHU2653-tipAp-ribo, pWHU2653-TRM-cas9, pWHU2653-TRM-cas9 and pWHU2653-TRMA-cas9, respectively. The homologous regions for HDR were amplified with primers 56 + 57 for the upstream arm and primers 58 + 59 for the downstream arm from *S. roseosporus* genome, and ligated into plasmids as described above. *cas9* was removed in the same way as above.

For plasmids with pSG5 *ori*, the *dptP* spacer was ligated to the *Bae*I site of pKC1139-TRM-cas9 and pKC1139-TRMA-cas9, respectively. The *dptP* upstream arm was amplified with primers 60 + 61, while the *dptP* downstream arms was amplified with primers 58 + 62, and fused to the *Eco*RI site of pKC1139-TRM-cas9 by Gibson assembly for pKC1139-TRM-cas9-dptP. For plasmid pKC1139-TRMA-cas9-dptP, the the *dptP* downstream arms was amplified with primers 58 + 63, and fused to *Eco*RI site of pKC1139-TRM-cas9 along with the upstream arm.

2 Supplementary Figures and Tables

2.1 Supplementary Figures

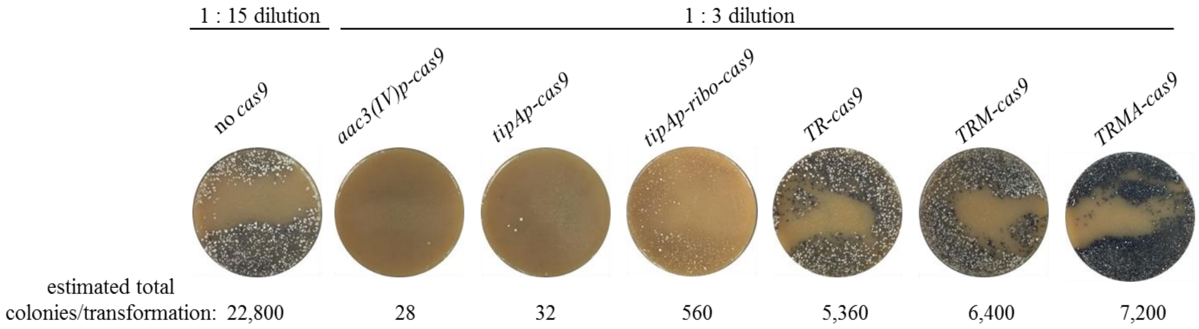

Figure S1. Estimation of transformation efficiency for *actII-ORF4* deletion in *S. coelicolor* with various combinatory controls of Cas9 activities based on the pIJ101 *ori* plasmid. The representative plates were photographed after one week of conjugation, and the estimated transformant numbers were shown below the plates. For the transformation without Cas9 (the first plate), the bacterial mixture was diluted in 1 : 15 and subjected to plating, while others were diluted in 1 : 3.

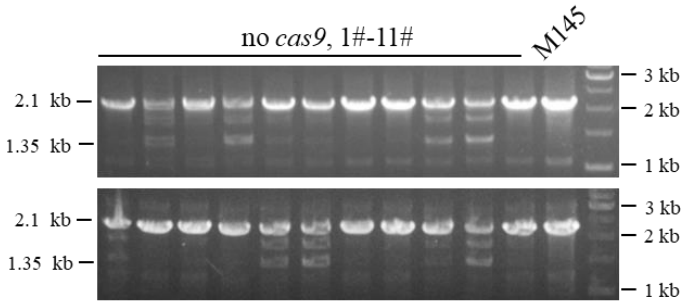

Figure S2. Confirmative PCR for deletion of *actII-ORF4* in *S. coelicolor* by CRISPR/Cas9 method based on pWHU2653 backbone but without Cas9, which was removed from the vector. Eleven transformants were validated by PCR as shown in Figure 2A.

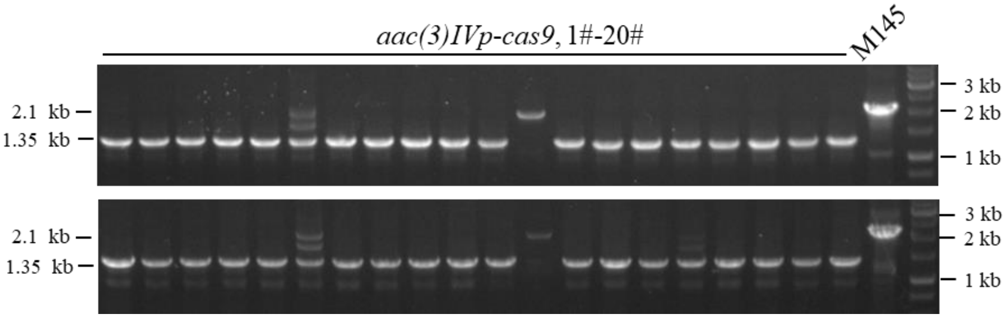

Figure S3. Confirmative PCR for deletion of *actII-ORF4* in *S. coelicolor* by CRISPR/Cas9 method based on pWHU2653 backbone. Cas9 expression was driven from the apramycin resistant gene promoter (*aac(3)IVp*). Twenty transformants were validated by PCR as shown in Figure 2A, and 90% (18/20) deletion rate was estimated.

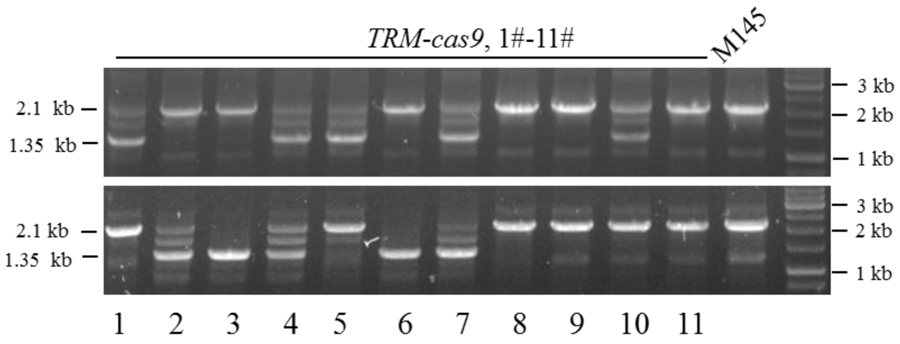

Figure S4. Confirmative PCR for deletion of *actII-ORF4* in *S. coelicolor* by CRISPR/Cas9 method based on pWHU2653 backbone. Cas9 was split expressed by fusion to pMag or nMag, and controlled under *tipAp* and riboswitch (*TRM-cas9*). Eleven transformants were validated by PCR as shown in Figure 2A.

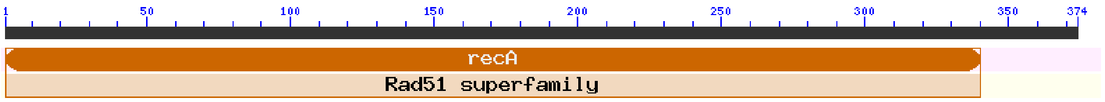

Figure S5. Domain organization of RecA from *S. coelicolor*.

```
1         MAGTDREKALDAALAQIERQFGKGAVMRMGDRTNEPIEVIPTGSTALDVALGVGGIPRGRVVEVYGPESSGKTTLT  76
1         MAGNDREKALDAALAQIERQFGKGAVMRLGERPNEPIEVIPTGSTALDVALGVGGLPRGRVVEVYGPESSGKTTLT  76
1         MAGTDREKALDAALAQIERQFGKGAVMRMGDRTQEPIEVISTGSTALDIALGVGGLPRGRVVEIYGPESSGKTTLT  76
1         MAGTDREKALDAALAQIERQFGKGAVMRLGERPNEPIEVIPTGSTALDVALGVGGLPRGRVVEVYGPESSGKTTLT  76
1         MAGTDREKALDAALAQIERQFGKGAVMRMGDRTNEPIEVIPTGSTALDVALGVGGIPRGRVVEVYGPESSGKTTLT  76
1    [43] MAGTDREKALDAALAQIERQFGKGAVMRMGDRTQEPIEIIPTGSTALDVALGVGGLPRGRVVEIYGPESSGKTTLT  119

77   LHAVANAQKAGGQVAFVDAEHALDPEYAKKLGVDIDNLILSQPDNGEQALEIVDMLVRSGALDLIVIDSVAALVPRAEIE  156
77   LHAVANAQKLGGSVAFIDAEHALDPEYAKKLGVDIDSLILSQPDNGEQALEIVDMLVRSGALDLIVIDSVAALVPRAEIE  156
77   LHAVANAQKAGGQVAFVDAEHALDPEYAKKLGVDIDNLILSQPDNGEQALEIVDMLVRSGALDLIVIDSVAALVPRAEIE  156
77   LHAVANAQKAGGQVAFVDAEHALDPEYAKKLGVDIDNLILSQPDNGEQALEIVDMLVRSGALDLIVIDSVAALVPRAEIE  156
77   LHAVANAQKAGGQVAFVDAEHALDPEYAKKLGVDIDNLILSQPDNGEQALEIVDMLVRSGALDLIVIDSVAALVPRAEIE  156
120  LHAVANAQRAGGQVAFVDAEHALDPEYARKLGVDIDNLILSQPDNGEQALEIVDMLVRSGALDLIVIDSVAALVPRAEIE  199

157  GEMGDSHVGLQARLMSQALRKITSALNQSKTTAIFINQLREKIGVMFGSPETTTGGRALKFYASVRLDIRRIETLKDGTD  236
157  GEMGDSHVGLQARLMSQALRKITSALNQSKTTAIFINQLREKIGVMFGSPETTTGGRALKFYASVRLDIRRIETLKDGTD  236
157  GEMGDSHVGLQARLMSQALRKITSALNQSKTTAIFINQLREKIGVMFGSPETTTGGRALKFYASVRLDIRRIETLKDGTD  236
157  GEMGDSHVGLQARLMSQALRKITSALNQSKTTAIFINQLREKIGVMFGSPETTTGGRALKFYASVRLDIRRIETLKDGTD  236
157  GEMGDSHVGLQARLMSQALRKITSALNQSKTTAIFINQLREKIGVMFGSPETTTGGRALKFYASVRLDIRRIETLKDGTD  236
200  GEMGDSHVGLQARLMSQALRKITSALNQSKTTAIFINQLREKIGVMFGSPETTTGGRALKFYASVRIDIRRIETLKDGTE  279

237  AVGNRTRVKVVKNKVAPPFKQAEFDILYGQGISREGGLIDMGVENGFVRKAGAWYTYEGDQLGQGKENARNFLKDNPDLA  316
237  AVGNRTRVKVVKNKVAPPFKQAEFDILYGQGISREGGLIDMGVEHGFVRKAGAWYTYEGDQLGQGKENARNFLKDNPDLA  316
237  AVGNRTRVKVVKNKVAPPFKQAEFDILYGQGISREGGLIDMGVEHGFVRKAGAWYTYEGDQLGQGKENARNFLKDNPDLA  316
237  AVGNRTRVKVVKNKVAPPFKQAEFDILYGQGISREGGLIDMGVENGFVRKAGAWYTYEGDQLGQGKENARNFLKDNPDLA  316
237  AVGNRTRVKVVKNKVAPPFKQAEFDILYGQGISREGGLIDMGVENGFVRKAGAWYTYEGDQLGQGKENARNFLKDNPDLA  316
280  AVGNRTRCKVVKNKVAPPFKQAEFDILYGQGISREGGLIDMGVEHGFVRKAGAWYTYESGQLGQGKENARNFLKDNPDLA  359

317  NEIEKKIKQKLGVGVHPEESATEPGADA----ASAAPADAAPAVPAPtTAKATK-SKATAAKS*  375
317  NEIEKKILEKLGVGVRPDADAGEPAADAA-AAAAPAADGAAKPAATS-AAKAKP-AKTAAAKS*  377
317  DEIERKIKEKLGVGVRPDAAKAEAATDAAAAADTAGTDDAAKSVPAP-ASKTAKaTKATAVKS*  379
317  NEIEKKIKEKLGVGVRPEEPTAEPGADAAVTSAAAATDDTAKTVSAP-AAKTTK-SKAAAAKS*  378
317  NEIEKKIKQKLGVGVHPEESATEPGADA----ASAAPADAAPAVPAPtTAKATK-SKAAAAKS-  374
360  DEIEKKIKDKLGVGVRTEAPGAEPGPDAAGAAP---GDEPAKAVPAP-ATK-SKaTRSTAAKS*  418
```

Figure S6. RecA is highly conserved in *Streptomyces*. Multiple alignment of RecA from six *Streptomyces* species (*S. coelicolor*, *S. griseus*, *S. venezuelae*, *S. avermitilis*, *S. lividans*, *S. clavuligerus*). Protein sequences were downloaded from StrepDB (http://strepdb.streptomyces.org.uk/), and alignment was performed on Multiple Alignment (https://blast.ncbi.nlm.nih.gov/Blast.cgi).

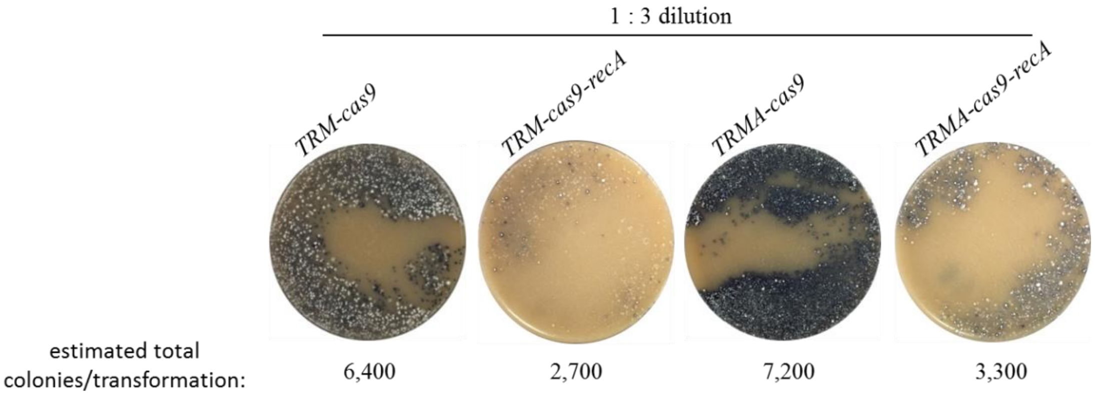

Figure S7. Estimation of transformation efficiency for *actII-ORF4* deletion in *S. coelicolor* with controlled Cas9 activity under *tipAp*, riboswitch and split pMag/nMag (*TRM-cas9*). *recA* and *atpD* were further expressed under *ermEp** alone or both. The bacterial mixtures for conjugation were all diluted in 1 : 3 and subjected to plating. The plates were photographed one week after conjugation, and the estimated transformants were shown below the plates.

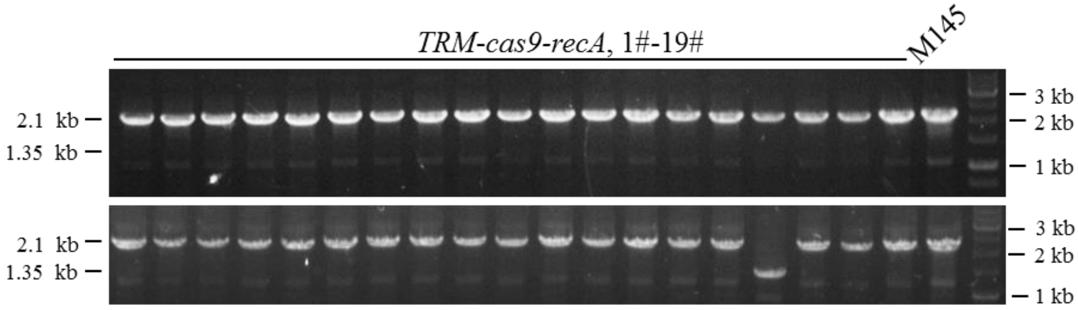

Figure S8. Confirmative PCR for deletion of *actII-ORF4* in *S. coelicolor* by CRISPR/Cas9 strategy based on pWHU2653 backbone. Cas9 was split expressed by fusion to pMag or nMag, controlled under *tipAp* and riboswitch, and *recA* was additionally expressed under *ermEp** (*TRM-cas9-recA*). Nineteen transformants were validated by PCR as shown in Figure 2A.

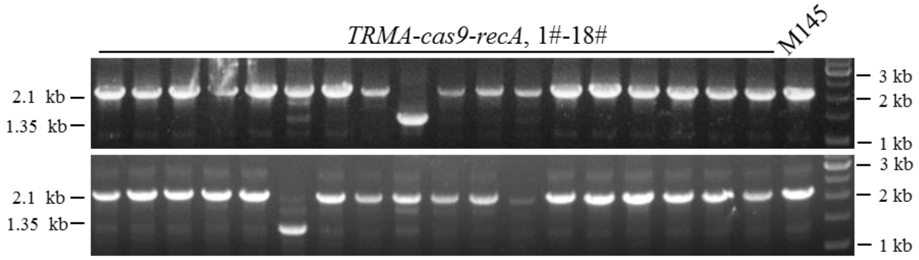

Figure S9. Confirmative PCR for deletion of *actII-ORF4* in *S. coelicolor* by CRISPR/Cas9 strategy based on pWHU2653 backbone. Both *recA* and *atpD* were additionally expressed under *ermEp** (*TRMA-cas9-recA*). Eighteen transformants were validated by PCR as shown in Figure 2A.

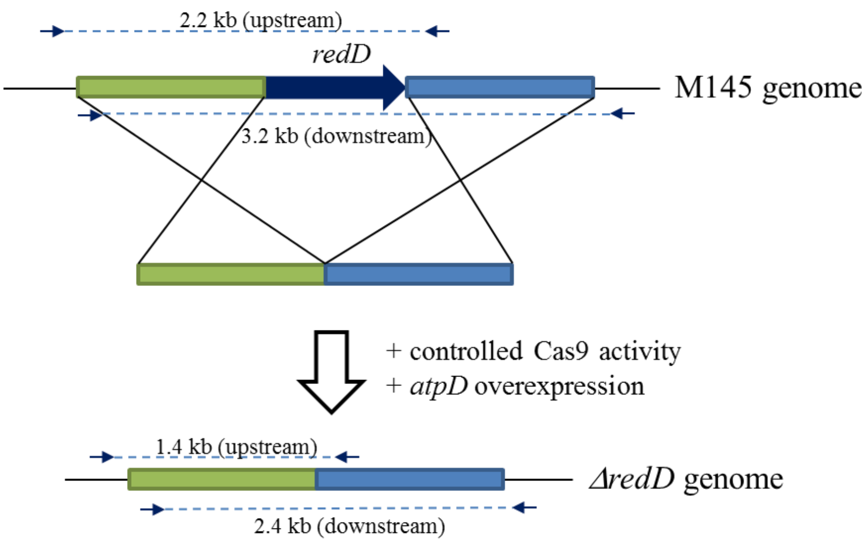

Figure S10. Schematic diagram of *redD* deletion in *S. coelicolor* by the CRISPR/Cas9 strategy-based homologous recombination. The primers for confirmative PCR were shown as arrows, and the estimated PCR products were also shown in dashed lines..

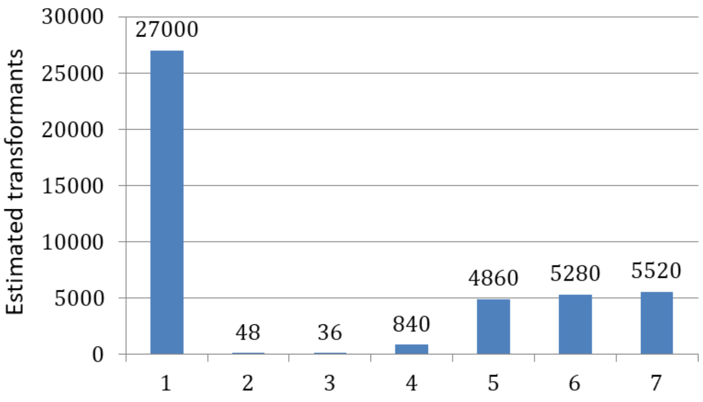

Figure S11. Estimated transformant numbers in *redD* deletion of *S. coelicolor* with engineered control of Cas9 activity and ATP supply as in Figure 2A. 1, no *cas9*; 2, *aac(3)IVp-cas9*; 3, *tipAp-cas9*; 4, *tipAp-ribo-cas9*; 5, *TM-cas9*; 6, *TRM-cas9*; 7, *TRMA-cas9*.

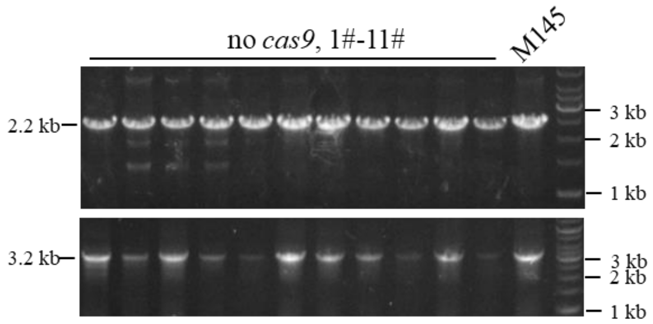

Figure S12. Confirmative PCR for deletion of *redD* in *S. coelicolor* by CRISPR/Cas9 method based on pWHU2653 backbone but without Cas9, which was removed from the vector. Eleven transformants were validated by PCR.

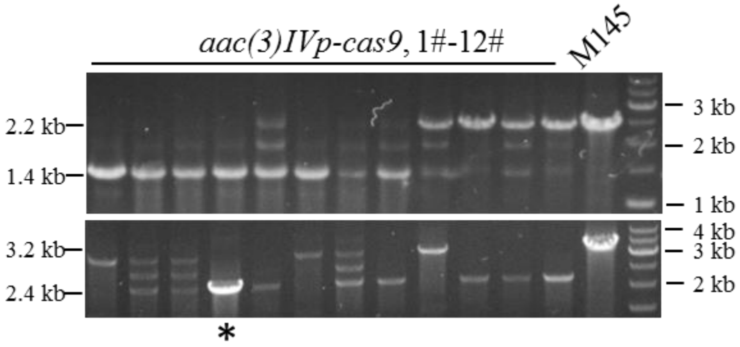

Figure S13. Confirmative PCR for deletion of *redD* in *S. coelicolor* by CRISPR/Cas9 method based on pWHU2653 backbone with Cas9 expressed under the *aac(3)IV* promoter. Twelve transformants were validated by PCR.

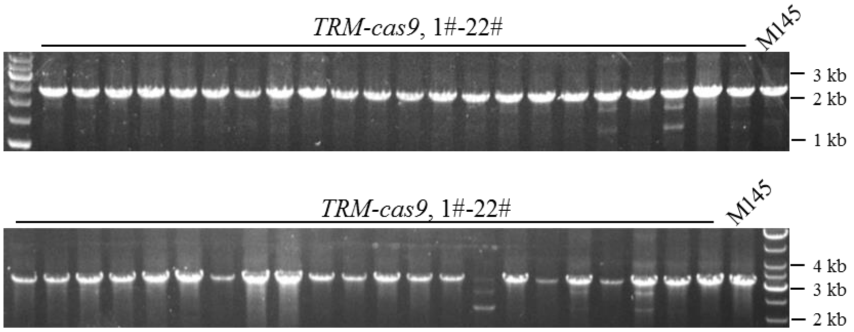

Figure S14. Confirmative PCR for deletion of *redD* in *S. coelicolor* by CRISPR/Cas9 method based on pWHU2653 backbone. Cas9 was split expressed by fusion to pMag or nMag, and controlled under *tipAp* and riboswitch (*TRM-cas9*). Twenty-two transformants were validated by PCR.

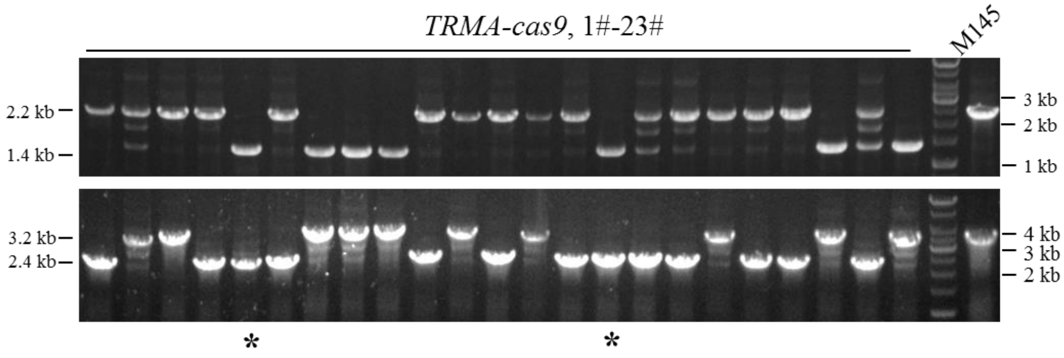

Figure S15. Confirmative PCR for deletion of *redD* in *S. coelicolor* by CRISPR/Cas9 strategy based on pWHU2653 backbone with ectopic expression of *atpD*. Cas9 was split expressed by fusion to pMag or nMag, and controlled under *tipAp* and riboswitch. *aptD* was further expressed under *ermEp** (*TRMA-cas9*). Twenty-three transformants were validated by PCR.

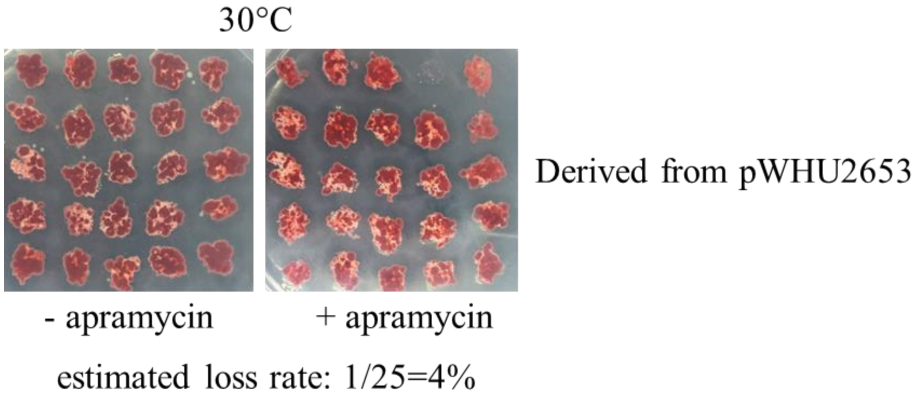

Figure S16. Estimation of loss rate of pWHU2653-derived plasmid. After confirmation of gene deletion, the strain containing plasmid pWHU2653-cas9 for *actII-ORF4* deletion was cultivated in TSB + 5% PEG6000 for 2 days at 30°C, and streaked on the R5 plate. Twenty-five single colonies were replicated in the R5 plate with or without apramycin for 4 days to determine the plasmid loss rate.

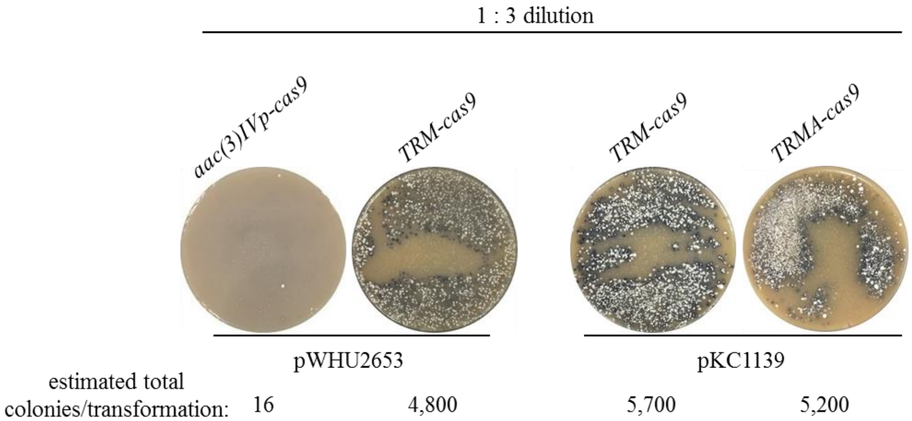

Figure S17. Estimation of transformation efficiency for *actII-ORF4* deletion in *S. coelicolor* with engineered controls of Cas9 activities as in Figure 2B, but based on the pSG5 *ori* plasmid. The representative plates were photographed after one week of conjugation, and the estimated transformant numbers were shown below the plates.

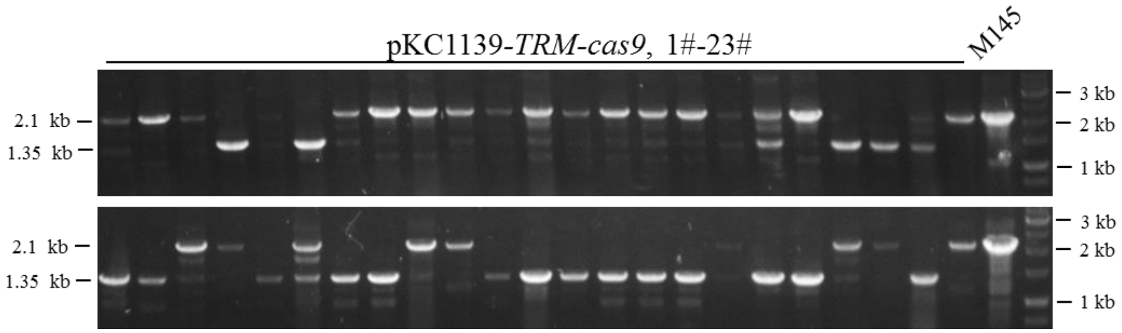

Figure S18. Confirmative PCR for deletion of *actII-ORF4* in *S. coelicolor* by CRISPR/Cas9 method based on pKC1139 backbone. Cas9 was split expressed by fusion to pMag or nMag, and controlled under *tipAp* and riboswitch (*TRM-cas9*). Twenty-three transformants were validated by PCR as shown in Figure 2A.

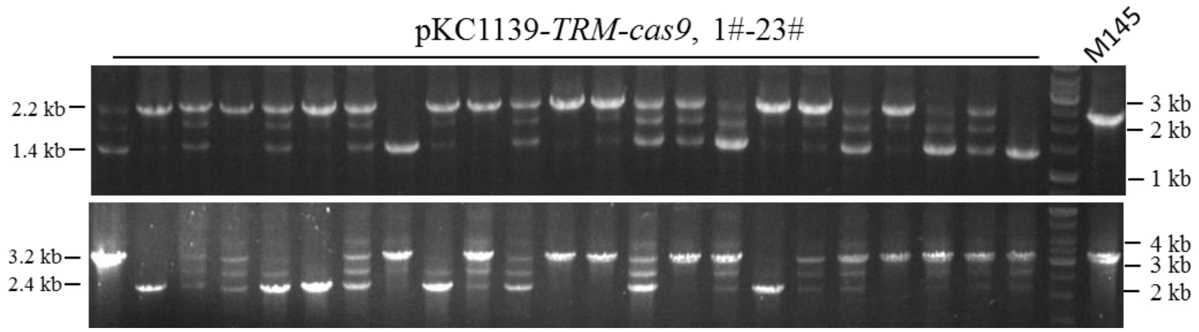

Figure S19. Confirmative PCR for deletion of *redD* in *S. coelicolor* by CRISPR/Cas9 method based on pKC1139 backbone. Cas9 was split expressed by fusion to pMag or nMag, and controlled under *tipAp* and riboswitch (*TRM-cas9*). Twenty-three transformants were validated by PCR.

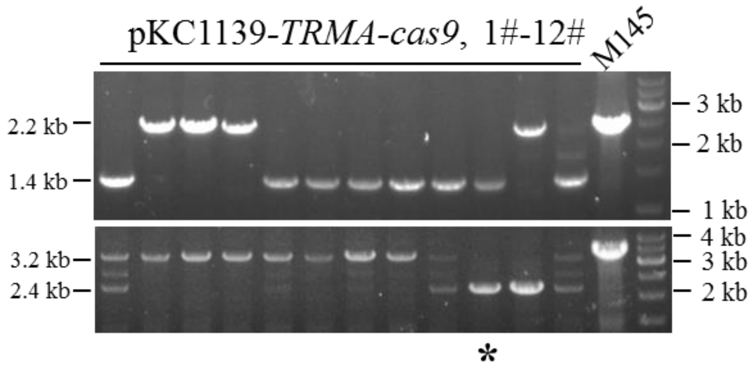

Figure S20. Confirmative PCR for deletion of *redD* in *S. coelicolor* by CRISPR/Cas9 strategy based on pKC1139 backbone with ectopic expression of *atpD*. Cas9 was split expressed by fusion to pMag or nMag, and controlled under *tipAp* and riboswitch. And *aptD* was further expressed under *ermEp** (*TRMA-cas9*). Twelve transformants were validated by PCR.

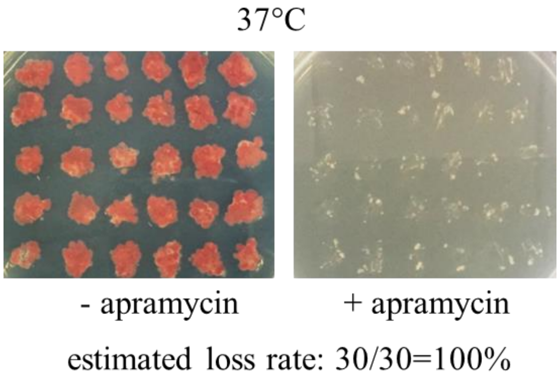

Figure S21. Estimation of loss rate of pSG5 *ori*-derived plasmids. After confirmation of *actII-ORF4* deletion, the strain containing plasmid pKC1139-TRMA-cas9 was cultivated in TSB + 5% PEG6000 for 2 days at 37°C, and streaked on the R5 plates. Thirty single colonies were replicated in the R5 plate with or without apramycin for 4 days to determine the plasmid loss rate.

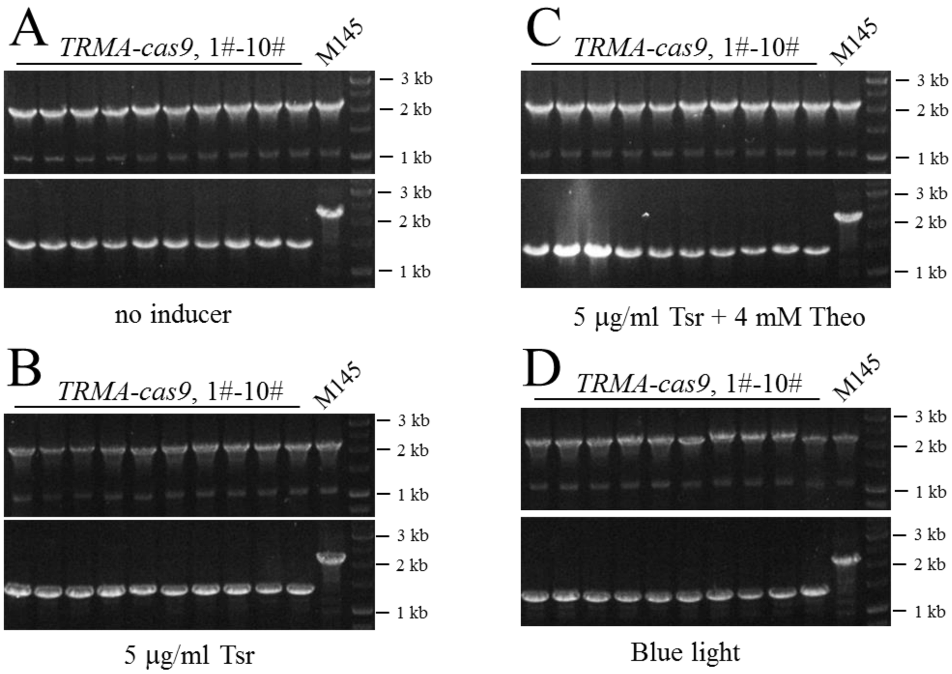

Figure S22. Effects of inducers thiostrepton (Tsr) and theophylline (Theo) for deletion of *actII-ORF4* in *S. coelicolor* containing single cross-over pKC1139-TRMA-cas9. Cells were cultured in TSB + 5% PEG6000 + 25 μg/ml apramycin for 2 days without inducers (A), with Tsr (B), with Theo (C), or with both Tsr and Theo (D), and streaked on R5 plates. Each of ten single colonies was confirmed by PCR as shown in Figure 2A.

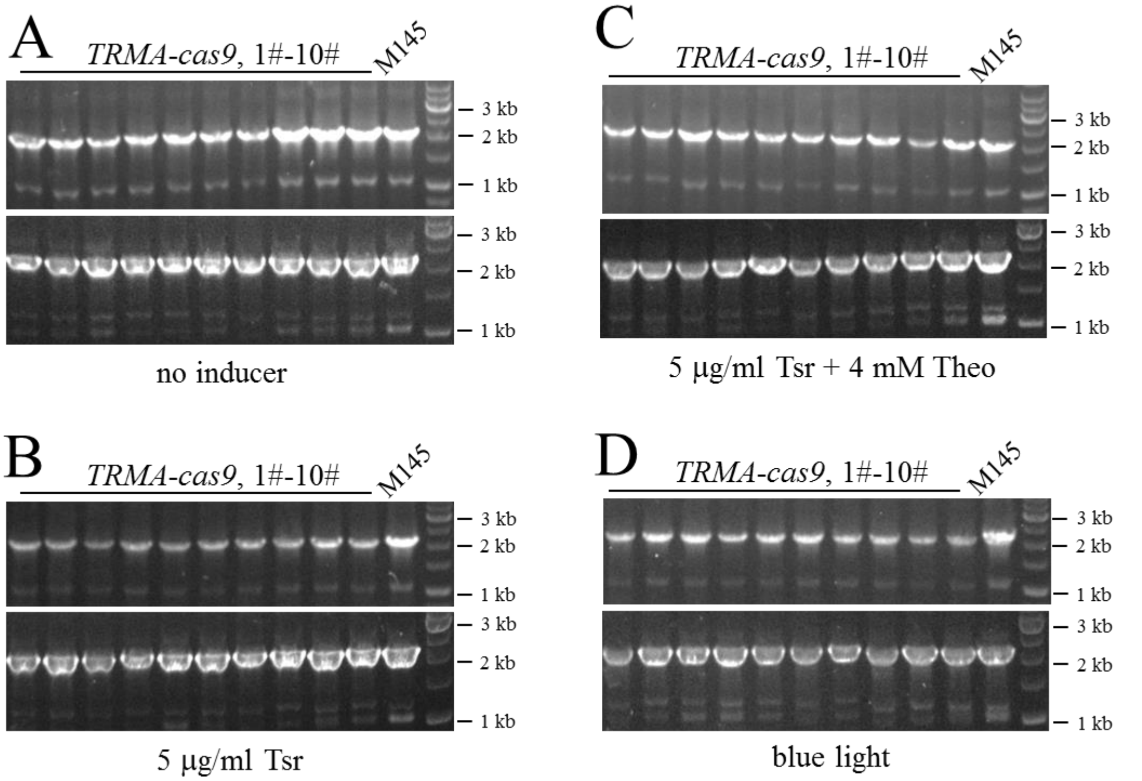

Figure S23. Effects of inducers thiostrepton (Tsr) and theophylline (Theo) for deletion of *actII-ORF4* in *S. coelicolor* containing replicative pKC1139-TRMA-cas9. Cells were cultured in TSB + 5% PEG6000 + 25 μg/ml apramycin for 2 days without inducers (A), with Tsr (B), with Theo (C), or with both Tsr and Theo (D), and streaked on R5 plates. Each of ten single colonies was confirmed by PCR as shown in Figure 2A.

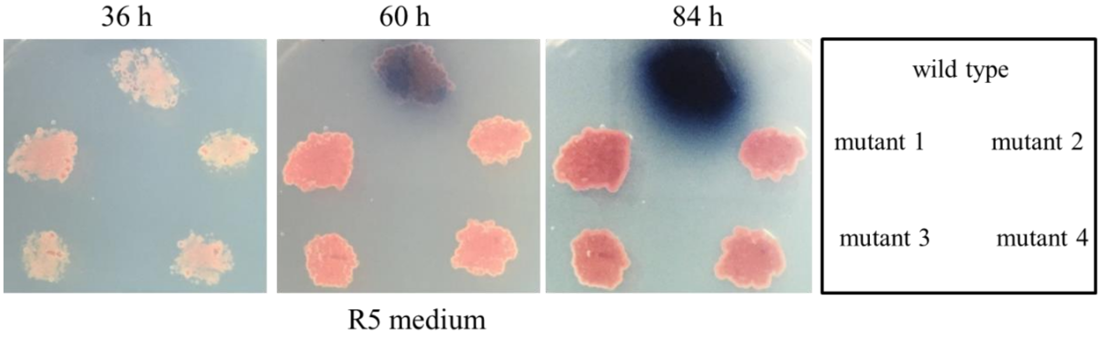

Figure S24. Phenotypic validation of Act production in *actII-ORF4* mutants (mutant 1-4). *S. coelicolor* wild type strain (M145), mutant 1 (from *aac3(IV)p-cas9*), mutant 2 (from pKC1139-based *TRMA-cas9*, induced), mutant 3 (from replicative pKC1139-based *TRMA-cas9*, combined induction) and mutant 4 (from single cross-over pKC1139-based *TRMA-cas9*, combined induction) were streaked on the R5 medium, incubated at 30°C for 36, 60 and 84 hours, respectively, and photographed.

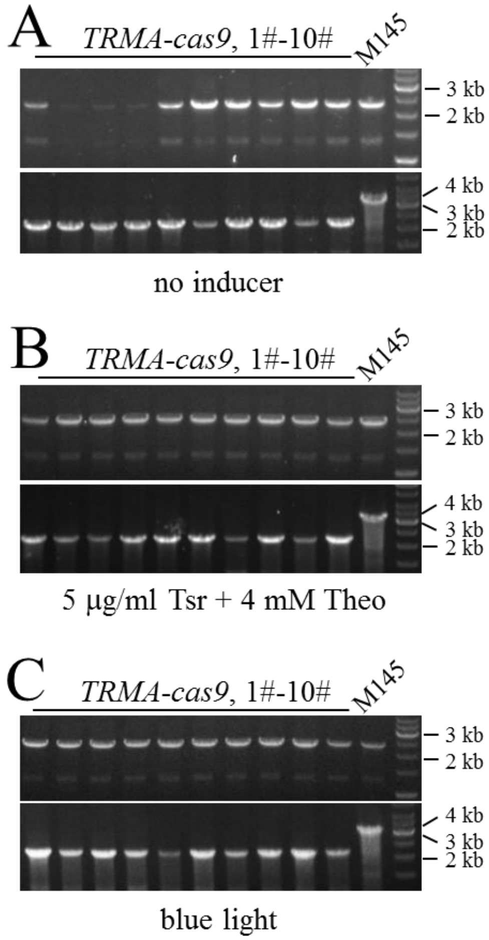

Figure S25. Effects of inducers for deletion of *redD* in *S. coelicolor* containing single cross-over pKC1139-TRMA-cas9. Cells were cultured in TSB + 5% PEG6000 + 25 μg/ml apramycin for 2 days without inducers (A), with Tsr + Theo (B) or only with blue light (C). Each of ten single colonies was confirmed by PCR.

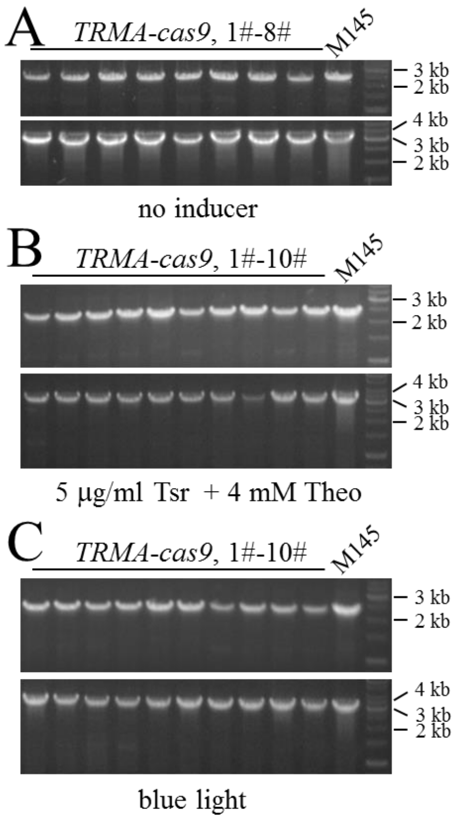

Figure S26. Effects of inducers for deletion of *redD* in *S. coelicolor* containing replicative pKC1139-TRMA-cas9. Cells were cultured in TSB + 5% PEG6000 + 25 μg/ml apramycin for 2 days without inducers (A), with Tsr + Theo (B) or only with blue light (C). Each of ten single colonies was confirmed by PCR.

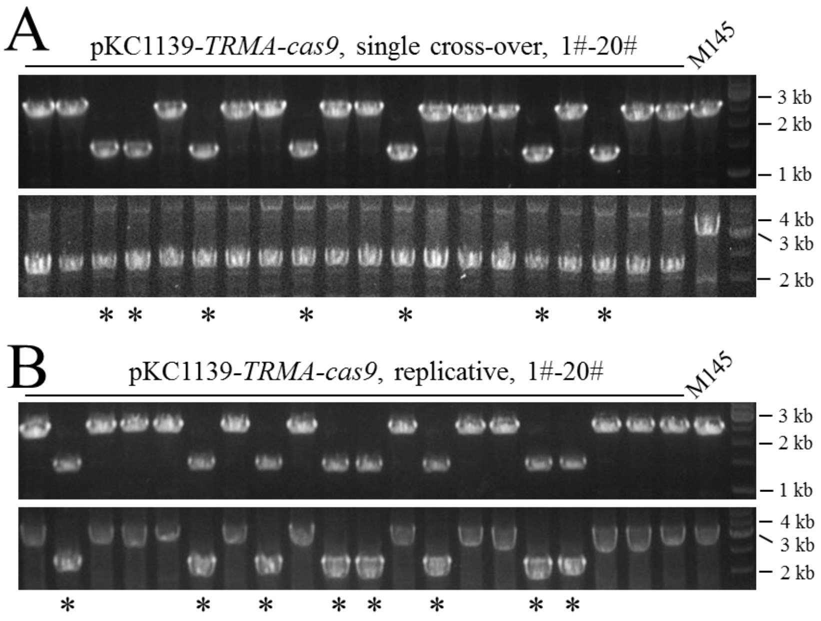

Figure S27. Effects of combined inducers (Tsr + Theo + blue light) for *redD* editing in *S. coelicolor* containing the single cross-over (A) or replicative (B) form of pKC1139-TRMA-cas9. Cells were cultured in TSB + 5% PEG6000 + 25 μg/ml apramycin for 2 days and induced for genome editing. Each of ten single colonies was confirmed by PCR.

A.

ggcggctccggctcgtccggcggcagcggccacaccctgtacgccccgggcggctacgacatcatgggctacctgcgccagatccgcaaccg
cccgaacccgcaggtcgagctgggccccgtcgacacctcctgcgccctgatcctgtgcgacctgaagcagaaggacaccccgatcgtctacgc
ctccgaggccttcctgtacatgaccggctactccaacgccgaggtcctgggccgcaactgccgcttcctgcagtccccggacggcatggtcaag
ccgaagtccacccggaagtacgtcgactccaacaccatcaacaccatgcgcaaggccatcgaccgcaacgccgaggtccaggtcgaggtcgt
caacttcaagaagaacggccagcgcttcgtcaacttcctgaccatgatcccggtccgcgacgagaccggcgagtaccgctactccatgggcttcc
agtgcgagaccgagtga

B.

GGSGSSGGSGHTLYAPGGYDIMGYLRQIRNRPNPQVELGPVDTSCALILCDLKQKDTPIVYA
SEAFLYMTGYSNAEVLGRNCRFLQSPDGMVKPKSTRKYVDSNTINTMRKAIDRNAEVQVEV
VNFKKNGQRFVNFLTMIPVRDETGEYRYSMGFQCETE

Figure S28. DNA sequence of pMag (A), which is chemically synthesized, and its deduced protein sequence (B).

A.

atgcacaccctgtacgccccgggcggctacgacatcatgggctacctggaccagatcggcaaccgcccgaacccgcaggtcgagctgggccc
cgtcgacacctcctgcgccctgatcctgtgcgacctgaagcagaaggacaccccgatcgtctacgcctccgaggccttcctgtacatgaccggct
actccaacgccgaggtcctgggccgcaactgccgcttcctgcagtccccggacggcatggtcaagccgaagtccacccggaagtacgtcgact
ccaacaccatcaacaccatgcgcaaggccatcgaccgcaacgccgaggtccaggtcgaggtcgtcaacttcaagaagaacggccagcgcttc
gtcaacttcctgaccatgatcccggtccgcgacgagaccggcgagtaccgctactccatgggcttccagtgcgagaccgagggcggctccggc
ggcagcggcggcggctcgggctccggctcgggcggc

B.

MHTLYAPGGYDIMGYLDQIGNRPNPQVELGPVDTSCALILCDLKQKDTPIVYASEAFLYMT
GYSNAEVLGRNCRFLQSPDGMVKPKSTRKYVDSNTINTMRKAIDRNAEVQVEVVNFKKNG
QRFVNFLTMIPVRDETGEYRYSMGFQCETEGGSGGSGGGSGSGSGG

Figure S29. DNA sequence of nMagHigh1 (nMag) (A), which is chemically synthesized, and its deduced protein sequence (B).

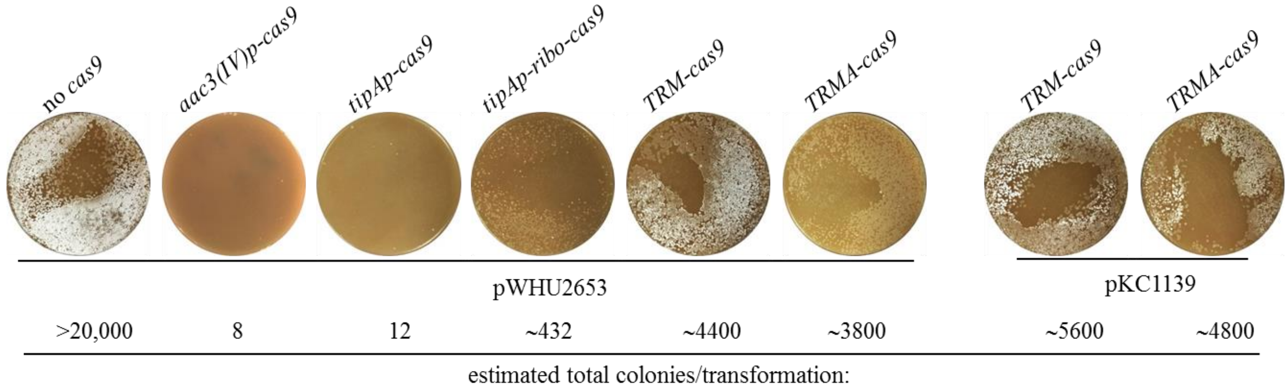

Figure S30. Estimation of transformation efficiency for *dptP* deletion in *S. roseosporus* with various combinatory controls of Cas9 activities based on the pIJ101 *ori* and pKC1139 pSG5 *ori* plasmid. The representative plates were photographed after one week of conjugation, and the estimated transformant numbers were shown below the plates.

2.2 Supplementary Tables

Table S1. Primers used in this study.

No.	Sequence (restriction sites are underlined)
1	GTGGTGGTGGTGGTGCTCGAGTGAATTCGATGGGGATCAAGGCGAA
2	CCGATGCTGTACTTCTTGTCCATATGTCCGCTCCCTTCTTCTCTG
3	GAAGAAGGGAGCGGACATAATACGACTCACTATAGGTTCCGGTGATACCAGCATC
4	CTTGTTGCCTCCTTAGCAGGGTGCTGCCAAGGGCATCAAGACGATGCTGGTATCACCG
5	CTGTACTTCTTGTCCATCTTGTTGCCTCCTTAGC
6	TGTACTGAGAGTGCACCATCTAGAGATTACTGTCGTTTAATG
7	CACCGCGGTGGCGGCCGCCATATGTCCGCTCCCTTCTTCTC
8	GAGAAGAAGGGAGCGGACATATGCACACCCTGTACGCCCC
9	GCCGCCCGAGCCGGAGCC
10	GGCTCCGGCTCGGGCGGCGATTCGGGCCAGGGCGACAGCC
11	ACCGCGGTGGCGGCCGCAGATCTTTAGTCGCCGCCCAGCTGGC
12	GAGAAGAAGGGAGCGGACATATGGACAAGAAGTACAGCATC
13	GAGCCGGAGCCGCCGATGACCTGGGCCTTCTGGATG
14	ATCGGCGGCTCCGGCTCGTC
15	TAGACACGTCTGAAGCTAGCCTCACTCGGTCTCGCACTG
16	TTCAGCGTGACATCATTCATAGGCGGCTTGCGCCCGATG

17	ATGTCCGCCTCCTTTGGTC
18	ACCAAAGGAGGCGGACATATGGCAGGAACCGACCGCGAG
19	GATCAAGGCGAATACTTCATATGATCCGTCTCGTACGGGG
20	GAGGCCCTTTCGTCTTCAAGGCGGCTTGCGCCCGATG
21	GGTCTCAACAGTGGTGGTCATATGTCCGCCTCCTTTGGTC
22	ATGACCACCACTGTTGAG
23	TTCGCCTTGATCCCCATCGCGACCAGCGCGACGTGC
24	ACAGCTATGACATGATTACGGCGGCTTGCGCCCGATG
25	TTCGCCTTGATCCCCATCGAATTCGCGACCAGCGCGACGTG
26	ATTACCAGGGACCGGAGTTCGTTTT
27	GAACTCCGGTCCCTGGTAATGCTGG
28	CGTGCTCGGCGGCTACCTGCGTTTT
29	GCAGGTAGCCGCCGAGCACGGCTGG
30	TGCTTGCGGCAGCGTGAAGCTTACCAAGCCGGAGTCGGTG
31	CTGCGCCCCCGTCGAGAT
32	ATCTCGACGGGGGCGCAGGAGAAGGTGCTCGTGTAG
33	CGACCTGCAGGCATGCAAGCTTGTGGTCGTCGTCATCGTC
34	GTGCTTGCGGCAGCGTGAAGCTTAGCAGCGAGACGACGAGG
35	TCCCCCACCCGTCATCC
36	GGATGACGGGTGGGGGAGCCGACACCAGCCTGCTC

37	CGACCTGCAGGCATGCAAGCTTGGTCGCCGTCCCGCTCCG
38	CTAGCCTGAAGCTTCAAG
39	AATTCTTGAAGCTTCAGG
40	CGCCTTGATCCCCATCGAATTCACCAAGCCGGAGTCGGTG
41	ACAGCTATGACATGATTACGTGGTCGTCGTCATCGTC
42	CTGCACGTCGCGCTGGTCGCGTGGTCGTCGTCATCGTC
43	CGCCTTGATCCCCATCGAATTCAGCAGCGAGACGACGAGG
44	TATGACATGATTACGAATTCGGTCGCCGTCCCGCTCC
45	CTGCACGTCGCGCTGGTCGGGTCGCCGTCCCGCTCC
46	GGGTCACCACCAGCCTG
47	GAGATGGTGGCGTACCTGA
48	CTATTGATTCGGAAGCCTCG
49	GCGACCTGTGAACGGTAG
50	CCCCAGGACTCGTTGAAG
51	GGTCGCCGTCCCGCTCCG
52	CGACGGGCAAACATCTG
53	GCAGGGCGCAGTCCAGG
54	CAGGATGGTGGAGAGCACGCGCTGG
55	GCGTGCTCTCCACCATCCTGGTTTT
56	CTGAGTGCTTGCGGCAGCGTGGCTTCTCCGCGTCGTC

57	GACCTGCAGGCATGCAAGCTTGGCAGCCCCGCTCTGTC
58	AGAGACAGAGCGGGGCTGCCCGCAGCTCGTGACCG
59	GACCTGCAGGCATGCAAGCTTCGGAGAACTCGTCACCAC
60	TTCGCCTTGATCCCCATCGCTTCTCCGCGTCGTCG
61	GGCAGCCCCGCTCTGTC
62	ATGACATGATTACGAATTCCGGAGAACTCGTCACCAC
63	TCGCGCTGGTCGCGAATTCCGGAGAACTCGTCACCAC

Table S2. Plasmids used in this study

Plasmid	Description	Reference
pWHU2653	*Streptomyces* CRISPR/Cas9 gene editing plasmid with pIJ101 *ori*, *aac(3)IVp-cas9*, *codA*	(1)
pET28a	*E. coli* expression vector	Novagen
pTA2	T-vector	Toyobo
pKC1139	*Streptomyces* temperature sensitive plasmid with pSG5 *ori*	(5)
pIJ8600	*E. coli-Streptomyces* shuttle vector with *tsr-to-tipAp* for thiostrepton inducible expression	(2)
pLM1	pIJ8630 with *ermEp**	(4)
pWHU2653-tipAp	Replace *aac(3)IVp* with *tipAp* for *cas9* expression in pWHU2653	This study
pWHU2653-tipAp-ribo	Insert theophylline-inducible riboswitch before *cas9* and after *tipAp* on pWHU2653-tipAp	This study
pWHU2653-TM-cas9	Cas9 is expressed as split fusions as NCas9-pMag and nMag-CCas9, both under *tipAp*	This study
pWHU2653-TRM-cas9	Insert riboswitch after *tipAp* on pWHU2653-TM-cas9	This study
pWHU2653-TRM-cas9-recA	*ermEp*-recA* on *Nde*I site of pWHU2653-TRM-cas9	This study
pWHU2653-TRMA-cas9	*ermEp*-atpD* on *Eco*RI site of pWHU2653-TRM-cas9	This study
pWHU2653-TRMA-cas9-recA	*ermEp*-atpD* on *Nde*I site of pWHU2653-TRM-cas9-recA	This study
pKC1139-TRM-cas9	Replace pIJ101 *ori* with pSG5 *ori*, no *codA*	This study

pKC1139-TRMA-cas9	*ermEp*-atpD* on *Eco*RI site of pKC1139-TRM-cas9	This study
pWHU2653-actII-ORF4	*actII-ORF4* spacer, homologous arms in pWHU2653 for *actII-ORF4* editing	This study
pWHU2653-actII-ORF4-cas9	Removal of *cas9* from pWHU2653-actII-ORF4, negative control	This study
pWHU2653-tipAp-actII-ORF4	*actII-ORF4* spacer, homologous arms in pWHU2653-tipAp for *actII-ORF4* editing	This study
pWHU2653-tipAp-ribo-actII-ORF4	*actII-ORF4* spacer, homologous arms in pWHU2653-tipAp-ribo for *actII-ORF4* editing	This study
pWHU2653-TRM-cas9-actII-ORF4	*actII-ORF4* spacer, homologous arms in pWHU2653-TRM-cas9 for *actII-ORF4* editing	This study
pWHU2653-TRMA-cas9-actII-ORF4	*actII-ORF4* spacer, homologous arms in pWHU2653-TRMA-cas9 for *actII-ORF4* editing	This study
pWHU2653-TRM-cas9-recA-actII-ORF4	*actII-ORF4* spacer, homologous arms in pWHU2653-TRM-cas9-recA for *actII-ORF4* editing	This study
pWHU2653-TRMA-cas9-recA-actII-ORF4	*actII-ORF4* spacer, homologous arms in pWHU2653-TRMA-cas9-recA for *actII-ORF4* editing	This study
pKC1139-TRM-cas9-actII-ORF4	*actII-ORF4* spacer, homologous arms in pKC1139-TRM-cas9 for *actII-ORF4* editing	This study
pKC1139-TRMA-cas9-actII-ORF4	*actII-ORF4* spacer, homologous arms in pKC1139-TRMA-cas9 for *actII-ORF4* editing	This study
pWHU2653-redD	*redD* spacer, homologous arms in pWHU2653 for *redD* editing	This study
pWHU2653-redD-cas9	Removal of *cas9* from pWHU2653-redD, negative control	This study

pWHU2653-tipAp-redD	*redD* spacer, homologous arms in pWHU2653-tipAp for *redD* editing	This study
pWHU2653-tipAp-ribo-redD	*redD* spacer, homologous arms in pWHU2653-tipAp-ribo for *redD* editing	This study
pWHU2653-TRM-cas9-redD	*redD* spacer, homologous arms in pWHU2653-TRM-cas9 for *redD* editing	This study
pWHU2653-TRMA-cas9-redD	*redD* spacer, homologous arms in pWHU2653-TRMA-cas9 for *redD* editing	This study
pWHU2653-TRM-cas9-recA-redD	*redD* spacer, homologous arms in pWHU2653-TRM-cas9-recA for *redD* editing	This study
pWHU2653-TRMA-cas9-recA-redD	*redD* spacer, homologous arms in pWHU2653-TRMA-cas9-recA for *redD* editing	This study
pKC1139-TRM-cas9-redD	*redD* spacer, homologous arms in pKC1139-TRM-cas9 for *redD* editing	This study
pKC1139-TRMA-cas9-redD	*redD* spacer, homologous arms in pKC1139-TRMA-cas9 for *redD* editing	This study
pWHU2653-dptP	*dptP* spacer, homologous arms in pWHU2653 for *dptP* editing	This study
pWHU2653-dptP-cas9	Removal of *cas9* from pWHU2653-dptP, negative control	This study
pWHU2653-tipAp-dptP	*dptP* spacer, homologous arms in pWHU2653-tipAp for *dptP* editing	This study
pWHU2653-tipAp-ribo-dptP	*dptP* spacer, homologous arms in pWHU2653-tipAp-ribo for *dptP* editing	This study
pWHU2653-TRM-cas9-dptP	*dptP* spacer, homologous arms in pWHU2653-TRM-cas9 for *dptP* editing	This study
pWHU2653-TRMA-cas9-dptP	*dptP* spacer, homologous arms in pWHU2653-TRMA-cas9 for *dptP* editing	This study

pKC1139-TRM-cas9- dptP	*dptP* spacer, homologous arms in pKC1139-TRM-cas9 for *dptP* editing	This study
pKC1139-TRMA-cas9-dptP	*dptP* spacer, homologous arms in pKC1139-TRMA-cas9 for *dptP* editing	This study

3 Supplementary References

1. Zeng H, Wen S, Xu W, He Z, Zhai G, Liu Y, et al. Highly efficient editing of the actinorhodin polyketide chain length factor gene in *Streptomyces coelicolor* M145 using CRISPR/Cas9-CodA(sm) combined system. *Appl Microbiol Biotechnol* (2015) 99(24):10575-85.

2. Sun JH, Kelemen GH, Fernandez-Abalos JM, Bibb MJ. Green fluorescent protein as a reporter for spatial and temporal gene expression in *Streptomyces coelicolor* A3(2). *Microbiol-UK* (1999) 145:2221-7.

3. Nihongaki Y, Kawano F, Nakajima T, Sato M. Photoactivatable CRISPR-Cas9 for optogenetic genome editing. *Nat Biotechnol* (2015) 33(7):755-60.

4. Mao XM, Zhou Z, Hou XP, Guan WJ, Li YQ. Reciprocal regulation between SigK and differentiation programs in *Streptomyces coelicolor*. *J Bacteriol* (2009) 191(21):6473-81.

5. Bierman M, Logan R, Obrien K, Seno ET, Rao RN, Schoner BE. Plasmid cloning vectors for the conjugal transfer of DNA from *Escherichia coli* to *Streptomyces* Spp. *Gene* (1992) 116(1):43-9.
